# News and Miscellany

**Published:** 1904-07

**Authors:** 


### News and Miscellany.

---

DR. DANIEL'S PAPER on "The Cause and Prevention of Rape; Sadism in the Negro," read at the recent meeting of the State Medical Association of Texas, at Austin, can be had in pamphlet form for 10 cents. The TEXAS MEDICAL JOURNAL, Austin, Texas.

THE Panhandle Medical Association held its "mid-summer meeting" at Amarillo ("the Queen City of the Plains"), July 12th, inst. Dr. D. R. Fly is President and Dr. W. H. Walker is Secretary. There was a large and enthusiastic gathering and an interesting program.

---

BACK NUMBERS WANTED.—I would be very glad to get a copy of each of the following back numbers, needed to complete the file of the library, Medical College, Galveston. I will give credit on subscription or pay 10 cents each, as preferred. I want Vol. 10, No. 6; Vol. 13, No. 5; Vol. 15, No. 11; Vol. 16, No. 2.

DANIEL.

---

WILL REPAIR YOUR ELECTRICAL MACHINES.—Static and all electrical medical apparatus put in running order. I am also agent for electrical and X-Ray apparatus. Oliver Brush, 710 Colorado Street, Austin, Texas.

---

GOOD LOCATION AVAILABLE..—For sale, a large fine residence, barn and stables, separate office near by. Seven acres of fine land. Price, $3000; half cash, balance to suit. Practice averages $1500 to $3000. Collections 98 per cent. Practice goes with property. Pretty village in a beautiful and rich farming section peopled with German and Bohemian settlers; prompt pay. Nearest competition ten miles. Dr. Breezes, care TEXAS MEDICAL JOURNAL.

---

A BARGAIN.—Five-room residence, barn, buggy house, orchard, good practice; situated in a fine farming country; collections good (95 per cent); no opposition. Nearest practicing physician seven miles. During the last two years cash collections amounted to $2000. Country settled mostly by Germans and Moravians. If you mean business, write to A. C. Mussil, Moravia, Texas.

---

A FINE OPENING for a good doctor. For $1200: a nice, modern six-room house on full block of ground, and a vacant lot in a nice town on the Central railroad, 41 miles from Houston. Has only one doctor; formerly had three to four. Address, Dr. J. D. Robinson, Ingram, Texas.

---

DEATH OF FATHER DAVIS.—Dr. N. S. Davis, through his lifelong interest in the American Medical Association, perhaps the best known physician in the country, died at his home in Chicago on June 16th, at the ripe age of 87 years. He was in good health up to within a few days of his death. He was born in Chenango county, N. Y., on January 9, 1817, and was graduated from the College of Physicians and Surgeons, Fairfield, N. Y., in 1837. He began practice in Vienna, N. Y., but soon removed to Binghamton, and in 1847 went to New York City, where he edited for a time the *Annalist*. In 1849 he received a call to the chair of physiology and pathology at Rush Medical College, Chicago, and moved to that city the latter part of August as soon as the subsidence of the cholera epidemic of that year left him to follow his own inclination. He continued to reside in Chicago until his death.

---

WHEN YOU WRITE to my advertisers, mention the "Red Back," please. It is money in my pocket.

---

THE TEXAS STATE BOARD OF MEDICAL EXAMINERS slew the Phillistines again at Houston last month. There were sixty-five applicants, of whom twenty-eight were rejected; over 40 per cent. Of the graduates of the Texas Medical College only one failed to pass.

---

DR. Q. A. SHUFORD, of Tyler, died in that city July 3d, inst., aged 87. Dear old Dr. Shuford, one of the best and kindest men, never appreciated at his real worth, except by those who knew him.

---

"THE DENVER ROAD" has just issued an illuminated "special announcement" that will be of interest and assistance to those who contemplate a trip to cool Colorado. They issue a "triangle, round-trip ticket to Colorado point (either going or returning via St. Louis), when reading in one direction via "The Denver Road" are good for ten days' stop-over in St. Louis; also unrestricted stop-over privileges in Colorado north of and including Trinidad, within their final limits. Write to A. A. Glisson, G. P. A., Fort Worth, Texas, for a copy. A postal card with get it.

---

WANTED.—Middle-aged physician for partner in well-established ethical office practice. No triflers need apply. Send self-addressed envelope. Address, Box 565, San Antonio, Texas.

---

PROF. A. JACOBI, at a mass meeting recently to working people, said: "The spread of tuberculosis is preventable by the people themselves. Attend to cleanliness and the laws of sanitation. Windows should be left open a little, not necessary more than a little in cold weather. Bathe frequently and stay in the open air as much as possible. In place of long trips to Colorado, short trips to farms near by will serve as well. There is plenty of pure bracing air near the city which would have a wonderful effect as a tonic in the incipient stages of the disease.'

---

BATTLE & Co. have just issued the second of the series of twelve illustrations of the intestinal parasites, and will send them free to physicians on application. Mention "Red Back."

---

THE AMERICAN NEUROLOGICAL ASSOCIATION has fixed the time of its meeting at St. Louis for September 15th, 16th, and 17th, and this will be immediately followed by the sessions of the various medical departments of the Congress of Arts and Sciences, beginning September 19th.

---

THE AMERICAN MEDICAL ASSOCIATION at the recent meeting at Atlantic City elected Dr. L. S. McMurtry, of Kentucky, President. and Dr. James Hall Bell, of San Antonio, Texas, First Vice-President.

---

℞ Young America
A toy pistol
The glorious 4th, aa, q. s.

M.: Result—very often tetanus. Pasteur Antitetanic Serum —we imported it only a few weeks ago from Paris—is at your disposal. No need for anyone to assure you that it's good serum. Right dose. Right price.

Very truly yours,
SHARP & DOHME.

---

FOR SALE.—Drug store and fixtures, well located on Main St., in flourishing Territory town of 6000. Address, J. R. J., lock box 785, Durant, I. T.

---

DISINFECT the discharges in typhoid fever or you will have other cases follow, also exclude the flies. An epidemic is sure to be followed for some time by sporadic cases from neglect of these precautions.

---

THE PASSING OF THE SANITARIAN.—Dr. A. N. Bell, the pioneer in sanitary science and for many years publisher and editor of *The Sanitarian,* Brooklyn, announces in the June number (Vol. LII, No. 415), that "with this issue *The Sanitarian, per se,* ceases. Beginning with the July issue, it is consolidated with the *Popular*

*Science Monthly,* Sub-station 84, New York City," to which all communications should be sent. The June number contains a portrait of the venerable and distinguished laborer in the cause of sanitation, now in his 84th year. I have framed it and given it a conspicuous place in my "Temple of Fame." It is very gratifying to note that the doctor—a living example of the correctness of the doctrines he so long inculcated by tongue and pen—is still in the enjoyment of strong physical and mental health, and that he promises an occasional contribution to the *P. S. Monthly,* successor to *The Sanitarian.* Dr. Bell will retire to his Prospect Park home and spend the remainder of his years amongst the blossoms and the bees, listening to the music of the song-birds. May his remaining years be unclouded, and serene and happy.

---

MARRIED, in Waco, Texas, June 15th ult., Dr. Frank Litten, a prominent practicing physician of Austin, to Miss Mildred Reese, of Waco.

---

THE HOST OF FRIENDS of Dr. T. J. Bennett, of Austin, Medical Councilor for the Ninth District, will be gratified to learn that at this writing he is fairly convalescent and will soon be able to be out. On the 12th of May last Dr. Bennett, while operating in a pus cavity, pricked his finger, from which violent blood poisoning resulted. Extensive suppuration along the lymph channels of the left arms occurred, necessitating several operations. For weeks he hovered between life and death; he passed through the valley of the shadow; but thanks to good nursing, skillful treatment, and his powerful *vis vitæ,* he pulled through, and it is hoped he will be spared to live many years of life, usefulness and happiness.

---

DR. JNO. T. HORTON, of Quanah, Texas, has removed to Austin and opened an office. He will enter his daughters at the University; that being the object in coming to Austin.

---

Medical students will be interested in the announcements of the Vanderbilt, the Tulane, the Jefferson, the Memphis, and the University of Nashville Medical colleges. See them.

---

OUR SPRIGHTLY CONTEMPORARY, the *Texas Courier-Record of Medicine,* complimenting the May edition of the "Red Back," says: * * * "The chief attraction of the issue, however, is the beautiful portrait of the Association's "Mascot," Mrs. Daniel. After looking at the picture, we neither wonder at the lady's receiving the unanimous vote of the society, nor that some of its members are voting yet; but we do wonder at some things. We look at the portrait of this most beautiful woman, then at that of the doctor, and we wonder—how it happened."

Dead easy! Rabbit's foot!

---

DR. F. D. THOMPSON, of Fort Worth, we learn from the *Texas Courier-Record of Medicine,* has reconsidered his acceptance of a chair in the Medical Department of the Baylor University (Dallas), notice of which acceptance was made in our June number, and will, after his return from his trip to Europe, "take up his customary work in connection with the Fort Worth University Medical College."

---

AT ATLANTIC CITY, American Medical Association meeting, the State Medical Association of Texas was represented by Prof. J. W. McLaughlin, M. D., Galveston; W. B. Russ, M. D., San Antonio, and R. W. Knox, Houston. A strong team!

---

THE *Colorado Medical Journal* does itself great credit in its March issue, which contains some 300 pages. It is a "Special Tuberculosis Number," and contains papers on consumption by well-known authors from Maine to California. Those who are interested in the subject would do well to get a copy. Price, paper, $1.00. No. 133 Colfax Avenue, Denver, Colo.

